# Fusarium oxysporum f. sp. *niveum* Pumilio 1 Regulates Virulence on Watermelon through Interacting with the ARP2/3 Complex and Binding to an A-Rich Motif in the 3′ UTR of Diverse Transcripts

**DOI:** 10.1128/mbio.00157-23

**Published:** 2023-03-01

**Authors:** Yizhou Gao, Xiaohui Xiong, Hui Wang, Yan Bi, Jiajing Wang, Yuqing Yan, Dayong Li, Fengming Song

**Affiliations:** a State Key Laboratory of Rice Biology, Ministry of Agriculture Key Laboratory of Biology of Crop Pathogens and Insects of Zhejiang Province, Institute of Biotechnology, Zhejiang University, Hangzhou, China; University of Melbourne

**Keywords:** actin-related protein 2/3 complex, *Fusarium oxysporum* f. sp. *nievum*, *Fusarium* wilt, Pumilio protein, virulence, watermelon

## Abstract

Fusarium oxysporum f. sp. *niveum* (*Fon*), a soilborne phytopathogenic fungus, causes watermelon Fusarium wilt, resulting in serious yield losses worldwide. However, the underlying molecular mechanism of *Fon* virulence is largely unknown. The present study investigated the biological functions of six *FonPUFs*, encoding RNA binding Pumilio proteins, and especially explored the molecular mechanism of *FonPUF1* in *Fon* virulence. A series of phenotypic analyses indicated that *FonPUFs* have distinct but diverse functions in vegetative growth, asexual reproduction, macroconidia morphology, spore germination, cell wall, or abiotic stress response of *Fon*. Notably, the deletion of *FonPUF1* attenuates *Fon* virulence by impairing the invasive growth and colonization ability inside the watermelon plants. FonPUF1 possesses RNA binding activity, and its biochemical activity and virulence function depend on the RNA recognition motif or Pumilio domains. FonPUF1 associates with the actin-related protein 2/3 (ARP2/3) complex by interacting with FonARC18, which is also required for *Fon* virulence and plays an important role in regulating mitochondrial functions, such as ATP generation and reactive oxygen species production. Transcriptomic profiling of *ΔFonPUF1* identified a set of putative FonPUF1-dependent virulence-related genes in *Fon*, possessing a novel A-rich binding motif in the 3′ untranslated region (UTR), indicating that FonPUF1 participates in additional mechanisms critical for *Fon* virulence. These findings highlight the functions and molecular mechanism of FonPUFs in *Fon* virulence.

## INTRODUCTION

Fusarium oxysporum is one of the most devastating fungal pathogens that causes vascular wilt in many economically important crops, such as banana, tomato, melon, and watermelon, worldwide (1). In the past 2 decades, significant progress has been made in understanding the molecular network of F. oxysporum pathogenicity using various infection model systems, specifically the tomato- or *Arabidopsis*-F. oxysporum pathosystems (2). The advent of genome sequencing and functional genomics tools has enabled us to explore the molecular mechanism and regulatory network of pathogenicity in F. oxysporum through the identification of key pathogenicity/virulence genes. Like other phytopathogenic fungi, F. oxysporum has evolved multiple strategies to evade and suppress the host plant innate immunity. A group of effectors in F. oxysporum, known as Secreted In Xylem (SIX), was identified from the xylem sap proteome of infected tomato plants (3, 4). Moreover, whole-genome sequencing and bioinformatics analyses identified a total of 62 candidate effector proteins in *Arabidopsis*-infecting F. oxysporum strain 5176 (5). Some effectors of F. oxysporum, such as SIX1, Foa1, Foa2, and Foa3, have been shown to target and suppress pattern-triggered immunity at an early infection stage (6). A rapid alkalinization factor, secreted by F. oxysporum, was found to accelerate the infection in host plants by elevating the pH (7). F. oxysporum also secretes cell wall-degrading enzymes, including polygalacturonases, pectate lyases, xylanases, and proteases, to facilitate its penetration and colonization within the host plant system (8). Various signaling proteins, such as cyclic AMP-protein kinase A, mitogen-activated protein kinase (MAPK) cascades, and G-protein subunits α/β have been reported to be required for F. oxysporum pathogenicity (1, 9). Three MAPKs, including Fmk1, Mpk1, and Hog1, have been revealed to play distinct but critical roles in F. oxysporum pathogenicity (10). Phosphatases, such as Ptc6 and Msg5, were shown to affect the MAPK cascades by regulating the phosphorylation status of Mpk1 and Fmk1, thereby regulating the infection-related signaling process and virulence in F. oxysporum (11, 12). F. oxysporum reprograms its transcriptional machinery during interactions with host plants by differentially regulating a distinct set of transcription factors, including Fow2, Sge1, PacC, Snt2, Fost12, HapX, Ftf1, Con7-1, FNR1, CTI6, and MeaB, which play critical roles in pathogenicity through regulating the expression of virulence-related genes (2, 13–20). However, a global understanding of the molecular network regulating F. oxysporum pathogenicity needs to be established.

Posttranscriptional regulation of gene expression is an important regulatory strategy to control the physiological and biochemical processes in phytopathogenic fungi (21). RNA-binding proteins (RBPs) regulate posttranscriptional gene expression by affecting RNA processing, including RNA modification, splicing, polyadenylation, capping, localization, translation, and stability (22). The Pumilio protein family (PUF) is a group of important RBPs in eukaryotes that can bind to the 3′-untranslated regions (UTRs) of the target mRNAs to regulate their stability and translation (23). The PUFs have several conserved consecutive RNA binding Pumilio repeats (24). The functions of PUFs have been studied extensively in various organisms, including worms, yeast, fungi, plants, and mammals (25, 26). In yeast, six PUF proteins, namely, Puf1 (Jsn1), Puf2, Puf3, Puf4, Puf5, and Puf6, have been shown to play important roles in the posttranscriptional regulation of target genes, and a number of PUFs-dependent mRNA targets have been identified (23, 27, 28). Puf1 and Puf2 preferentially bind to mRNAs encoding membrane-associated proteins, while Puf3 mainly binds to cytoplasmic mRNAs encoding mitochondrial proteins (29). Puf4 and Puf5 target nuclear components-encoding mRNAs, such as nucleolar rRNA-processing factors, spindle pole body components, and several chromatin modifiers (29).

At the molecular level, posttranscriptional repression of target genes is the quintessential function of PUF proteins (30, 31). Accumulating evidence suggests that PUF proteins bind to specific regulatory elements in their target mRNAs, thereby leading to mRNA degradation and posttranscriptional repression (32–35). Yeast ScPUF4 and ScPUF5 recruit the CCR4-POP2-NOT complex to mediate the target mRNAs degradation via deadenylation (36). ScPUF3 interacts with Dhh1 to modulate the posttranscriptional modification of target mRNAs (36), while ScPUF6 regulates *ASH1* mRNA expression by interacting with the general translation factor Fun12 or with ScLoc1 and She2 (37–39). The biological functions of PUF proteins in different organisms have been elucidated (40). A diverse range of PUF protein targets has suggested their additional physiological roles crucial for yeast viability and development (29, 41). In yeast, PUF proteins have been shown to play critical roles in hyphal morphogenesis, mating-type switching, mitochondrial biogenesis, motility, and abiotic stress response (42–44); however, their biochemical activity and biological functions in plant-pathogenic fungi remain elusive.

Yeast ScJsn1 (ScPUF1) is a mitochondrial outer membrane protein that associates with the actin-related protein 2/3 (ARP2/3) complex to drive multiple imperative mitochondrial functions (45). The ARP2/3 complex regulates the actin nucleation and assembly (46) and contains seven subunits, including two core subunits ARP2 and ARP3 (47). It has been reported that Schizosaccharomyces pombe SpArc3 is required for polarity and endocytosis (48). The deletion of Candida albicans *Arc18* led to impaired adherence and reduced biofilm formation, revealing the critical roles of the ARP2/3 complex components in cell wall remodeling (49). However, the function of the ARP2/3 complex and its association with PUF1 in plant-pathogenic fungi need to be explored.

F. oxysporum f. sp. *niveum* (*Fon*) causes watermelon Fusarium wilt, leading to a significant reduction in production under severe infection conditions. However, little is known regarding the molecular mechanism of *Fon* pathogenicity. It was found that FonSIX6 (an effector protein), FonNot2 (a subunit of the carbon catabolite repression4-negative on TATAless complex), and FonNst2 (a nucleotide sugar transporter), are required for *Fon* virulence (50–52). The present study aimed to explore the function of PUF proteins in *Fon*, especially the molecular network regulating its virulence. Among the six *FonPUF* genes identified, FonPUF1 was found crucial for *Fon* virulence on watermelon. Moreover, biochemical assays established that FonPUF1 regulates diverse mitochondrial functions via interacting with the mitochondria-associated ARP2/3 complex. FonPUF1 was found to bind to the canonical motifs and a newly identified A-rich motif in the 3′ UTRs of mRNAs of upregulated differentially expressed genes (DEGs) in *ΔFonPUF1*. These findings indicate that FonPUF1 regulates *Fon* virulence on watermelon through interaction with the ARP2/3 complex and binding to a conserved A-rich motif in the 3′ UTR of diverse transcripts, thus providing new insights into the molecular mechanism and regulatory network of pathogenicity in plant-pathogenic fungi, especially in F. oxysporum.

## RESULTS

### Identification of *FonPUFs* in *Fon*.

BLASTp searching against the F. oxysporum f. sp. *lycopersici* genome database using yeast PUF proteins as queries identified 6 putative PUF-encoding genes, including *FOXG_01859*, *FOXG_02224*, *FOXG_07983*, *FOXG_00799*, *FOXG_01770*, and *FOXG_00261*, in F. oxysporum (see Table S1 in supplemental material). The predicted open reading frame (ORF) sequences of the identified *PUF* genes were amplified from *Fon* and named consecutively as *FonPUF1*-*6* (see Table S1). However, we could not amplify *FonPUF3* and *FonPUF4* ORF sequences. FonPUF proteins contain a conserved Pumilio domain with different numbers of Pumilio repeats, e.g., FonPUF1 and FonPUF2 contain 6 and 7 Pumilio repeats, respectively, FonPUF3 and FonPUF4 possess 8 repeats, while FonPUF5 and FonPUF6 harbor 5 Pumilio repeats (see Fig. S1A). Interestingly, in addition to the Pumilio domain, FonPUF1 contains another conserved RNA recognition motif (RRM) (see Fig. S1A), which is similar to the yeast ScPUF1 and ScPUF2 (28, 41). Phylogenetic analysis revealed that FonPUF1 is closely related to ScPUF1 (ScJsn1) and ScPUF2, showing 42% and 36% sequence identity, respectively (see Fig. S1B). Further, BLASTp searching identified orthologues of ScPUF1 and ScPUF2 in Magnaporthe. oryzae and Fusarium graminearum (see Fig. S1B and S2). In the phylogenetic tree, FonPUF2 and FonPUF3 were clustered with ScPUF3 and FonPUF4 with ScPUF4, while FonPUF5 and FonPUF6 were grouped with ScPUF6 (see Fig. S1B). Overall, the strong relationship among FonPFUs with other orthologues reveals their evolutionary and functional convergence among different fungi.

10.1128/mbio.00157-23.1 FIG S1 Protein characteristics of FonPUFs. (A and B) Schematic diagram (A) of protein domain structures and phylogenetic tree (B) of PUFs in Fusarium oxysporum f. sp. *niveum* (*Fon*), Saccharomyces cerevisiae (*Sc*), Fusarium graminearum (*Fg*), and Magnaporthe oryzae (*Mg*). SMART protein database (http://smart.embl-heidelberg.de/) and Basic Local Alignment Search Tool of the NBCI protein database (https://blast.ncbi.nlm.nih.gov/Blast.cgi) were used for protein sequence retrieval and protein domain analysis. The phylogenetic tree was constructed via the MEGA7 software using the neighbor-joining method. Download FIG S1, EPS file, 7.2 MB. Copyright © 2023 Gao et al. 2023 Gao et al. https://creativecommons.org/licenses/by/4.0/ This content is distributed under the terms of the Creative Commons Attribution 4.0 International license.

10.1128/mbio.00157-23.2 FIG S2 Sequence alignment of FonPUFs with orthologues from other fungi. Alignment was performed by CLUSTALW program using amino acid sequences of FonPUFs and their orthologues. Pumilio repeat is boxed with red lines. Download FIG S2, EPS file, 7.7 MB. Copyright © 2023 Gao et al. 2023 Gao et al. https://creativecommons.org/licenses/by/4.0/ This content is distributed under the terms of the Creative Commons Attribution 4.0 International license.

10.1128/mbio.00157-23.8 TABLE S1 Characteristics of the *FonPUF* genes and proteins in Fusarium oxysporum f. sp. *niveum*. Download Table S1, XLSX file, 0.01 MB. Copyright © 2023 Gao et al. 2023 Gao et al. https://creativecommons.org/licenses/by/4.0/ This content is distributed under the terms of the Creative Commons Attribution 4.0 International license.

### Generation of targeted disruption or knockdown mutants for *FonPUFs*.

To investigate the functions of *FonPUF1*-*6* in *Fon*, deletion mutants for each target gene were generated using the homologous recombination strategy (see Fig. S3A) and named *ΔFonPUF1*, *ΔFonPUF2*, *ΔFonPUF3*, *ΔFonPUF4*, and *ΔFonPUF6*. The deletion mutants were further confirmed by Southern blotting via detecting a 679-bp hygromycin (*HPH*) fragment (see Fig. S3B). Reverse transcriptase-quantitative PCR (RT-qPCR) revealed that the transcript level of the *FonPUFs* in their corresponding deletion mutants was undetectable (see Fig. S3C). Despite three independent attempts, we failed to obtain a deletion mutant for *FonPUF5* and thus generated *FonPUF5*-RNAi strain using pSilent1 plasmid system. The transcript level of *FonPUF5* in *FonPUF5*-RNAi strain was <25% of that in wild type (WT) (see Fig. S3C).

10.1128/mbio.00157-23.3 FIG S3 Generation and validation of *FonPUFs* and *FonARC18* targeted disruption mutants and *FonARP3-*RNAi strains. (A) Strategy for generation of FonPUF-targeted disruption mutant strains. *HPH*, hygromycin B resistance gene cassette. The *HPH* fragment used as a probe for hybridization is indicated. (B) Validation of *FonPUFs* deletion mutants by Southern blotting. Genomic DNA was digested with different restriction enzymes as indicated. (C) Expression levels of *FonPUFs* in WT, targeted disruption, and RNAi mutants. ND, not detectable. (D) Strategies for generation of targeted disruption mutant for *FonARC18*. (E) Southern blotting of the deletion mutant *ΔFonARC18*. *HPH* fragment indicated in panel D was used as the probe to characterize *ΔFonARC18*. Genomic DNA was digested with different restriction enzymes as indicated. (F) Relative expression levels of *FonARP3* in *FonARP3*-RNAi strains. The fragment in ORF of *FonARP3* was cloned into the MCS1 and MCS2 region of pSlient1 vector. The relative expression level of *FonARP3* in WT strain was set to 1. RT-qPCR data in panels C and F were normalized by using the *FonActin* as an internal reference. Each experiment was repeated three times independently. Data presented here are the means ±SD, and asterisks above the columns indicate the significant difference at *P < *0.05 level. Download FIG S3, EPS file, 7.5 MB. Copyright © 2023 Gao et al. 2023 Gao et al. https://creativecommons.org/licenses/by/4.0/ This content is distributed under the terms of the Creative Commons Attribution 4.0 International license.

### *FonPUFs* differentially regulate the growth and development of *Fon*.

**FIG 1:**
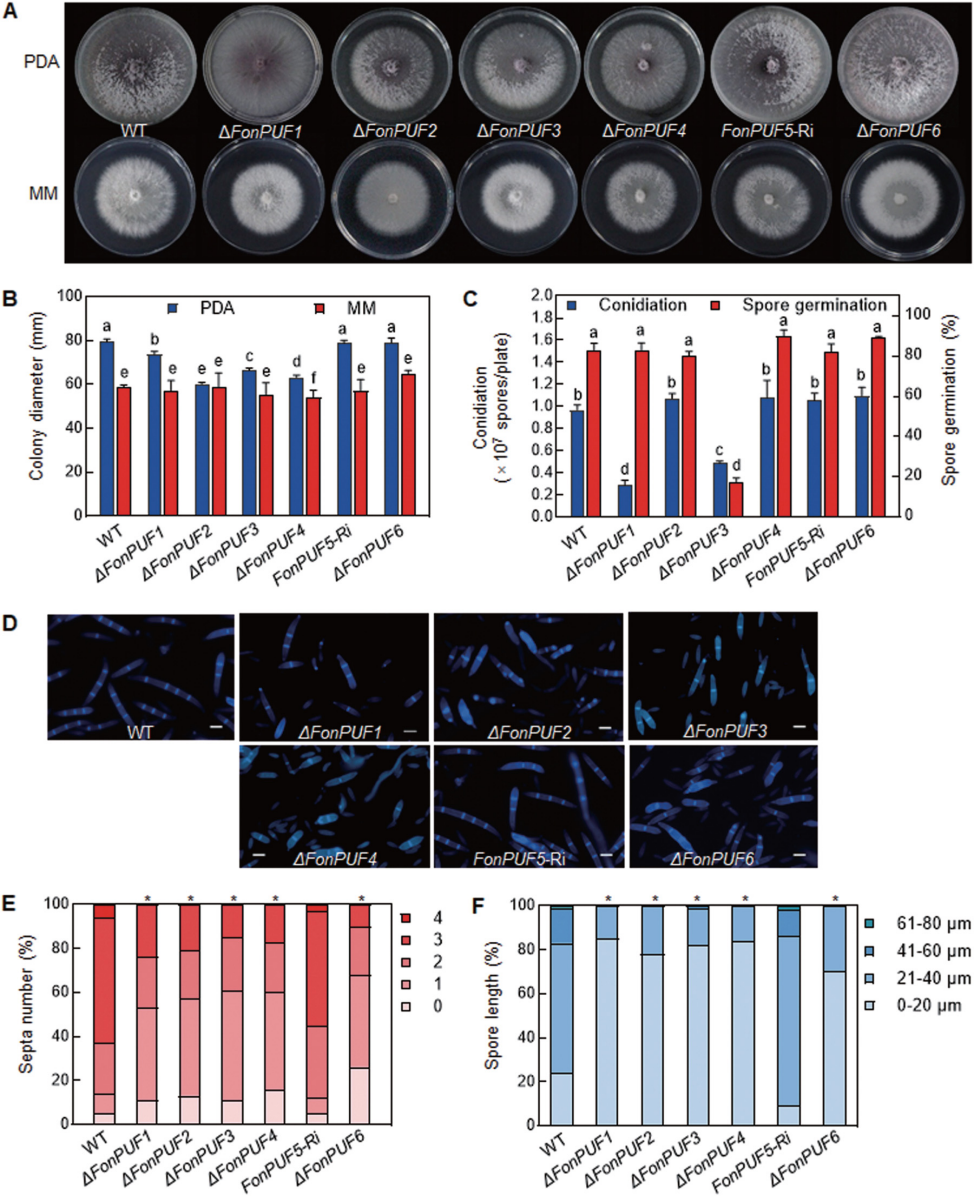
Involvement of *FonPUFs* in regulating vegetative growth, conidiation, and spore germination in *Fon*. (A and B) Colony morphology (A) and colony diameter (B) of WT and *ΔFonPUFs* deletion mutants on PDA and MM at 7 days postincubation. (C) Macroconidia production and spore germination of WT and *ΔFonPUFs* deletion mutant strains. (D) Morphology of CFW-stained macroconidia of WT and *ΔFonPUFs* deletion mutants. Scale bar = 5 μm. (E and F) Septa number (E) and length (F) of macroconidia of WT and *ΔFonPUFs* deletion mutants. Experiments in panels A and D were independently performed three times with similar results. Data presented in panels B and C are the means ± SD from three independent experiments, and different letters above columns indicate statistically significant difference at *P < *0.05 level calculated by LSD test. Data in panels E and F annotated with asterisks are significantly different from the WT (*P < *0.05).

The mutants *ΔFonPUF1*, *ΔFonPUF2*, *ΔFonPUF3*, and *ΔFonPUF4* showed a lower growth rate than WT on potato dextrose agar (PDA) medium, while only *ΔFonPUF4* showed significantly reduced mycelial growth in comparison to WT on the minimal medium (MM) (Fig. 1A and B). In mung bean liquid (MBL) medium, *ΔFonPUF1* and *ΔFonPUF3* produced significantly fewer macroconidia than WT (Fig. 1C). In spore germination assays, only *ΔFonPUF3* showed a significant reduction in conidial germination, compared to WT and other *ΔFonPUFs* (Fig. 1C). The mutants produced morphologically different macroconidia with fewer septa and shorter length than WT (Fig. 1D to F). Precisely, most of the macroconidia produced by the mutants had less than three septa, while most of WT macroconidia had three or more septa (Fig. 1E). Taken together, these data suggest that FonPUFs independently regulate mycelial growth, macroconidia morphology, asexual reproduction, and spore germination, thus playing distinct functions in the growth and development of *Fon*.

### *FonPUFs* play distinct roles in cell wall and abiotic stress response of *Fon*.

**FIG 2:**
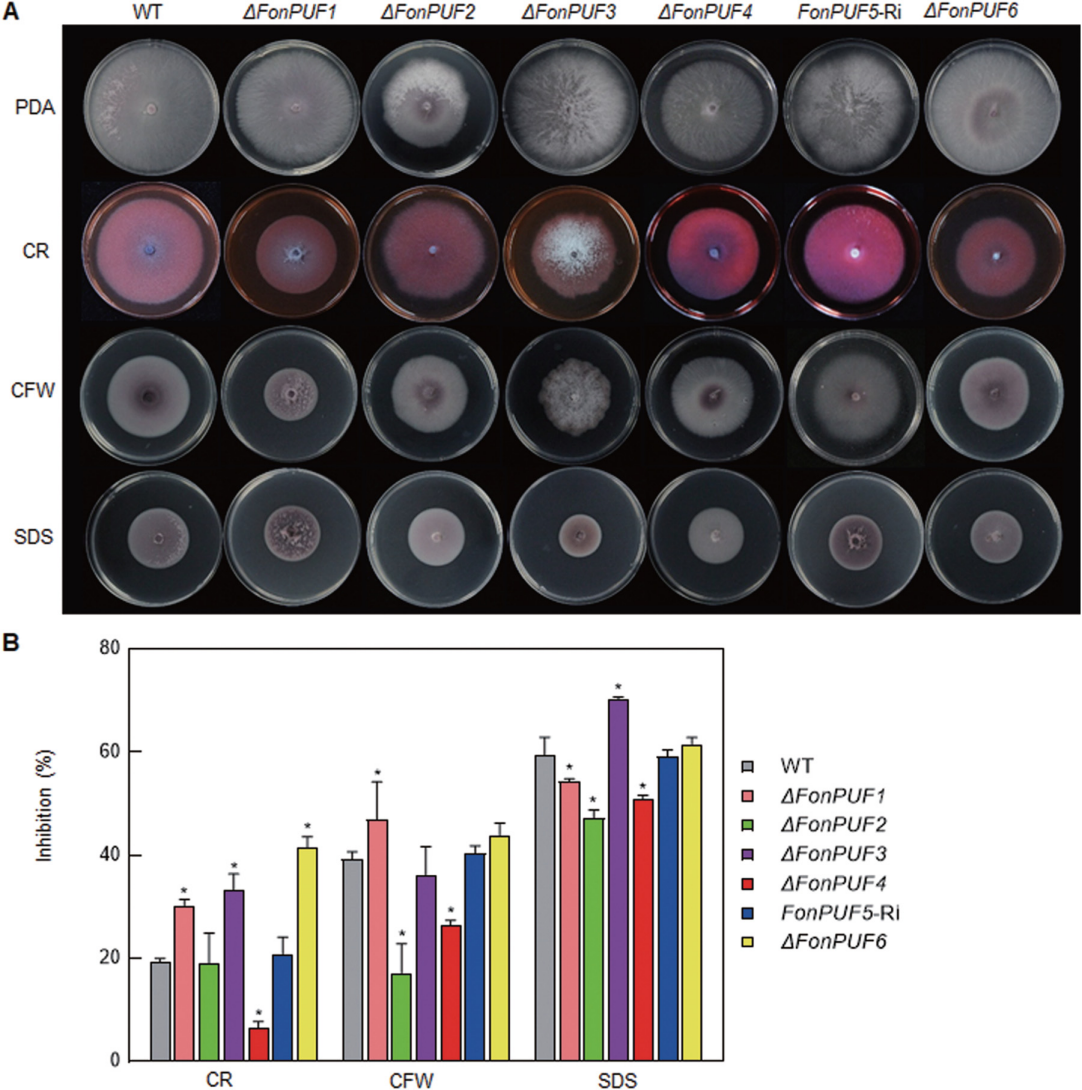
Involvement of *FonPUFs* in cell wall stress response of *Fon*. (A and B) Sensitivity (A) and mycelial growth inhibition rate (B) of WT and *ΔFonPUFs* deletion mutants in response to different cell wall-perturbing agents. All strains were grown on PDA amended with 0.02% CR, 0.02% CFW, or 0.03% SDS at 26°C for 7 days. Experiments in panel A were independently performed three times with similar results, and data presented in panel B are the means ± SD from three independent experiments. Data in panel B annotated with asterisks are significantly different from WT (*P < *0.05).

To investigate the involvement of *FonPUFs* in environmental stresses, the mycelial growth of the mutants was compared with WT in the presence of different cell wall perturbing agents, such as Congo red (CR), calcofluor white (CFW) or sodium dodecyl sulfate (SDS), and abiotic stress-inducing reagents, including sodium chloride (NaCl), sorbitol, calcium chloride (CaCl_2_), and magnesium chloride (MgCl_2_). Overall, the mutants showed differential sensitivity to cell wall perturbing agents (Fig. 2). Specifically, *ΔFonPUF1* became more sensitive to CR and CFW but was more resistant to SDS. *ΔFonPUF2* was more resistant to CFW and SDS, while *ΔFonPUF3* was more sensitive to CR and SDS. *ΔFonPUF4* showed tolerance to all the tested cell wall perturbing agents, while *ΔFonPUF6* exhibited increased CR sensitivity. Similarly, the mutants also displayed distinct sensitivity to different osmotic and ionic stressors (see Fig. S4). Under NaCl stress, the growth inhibition rate of *ΔFonPUF1* was obviously increased, while *ΔFonPUF2*, *ΔFonPUF3*, *ΔFonPUF4*, and *ΔFonPUF6* showed less growth inhibition. Moreover, *ΔFonPUF1*, *ΔFonPUF2*, and *ΔFonPUF6* exhibited higher sensitivity to sorbitol, whereas *ΔFonPUF2*, *ΔFonPUF3*, and *ΔFonPUF4* showed different levels of sensitivity to CaCl_2_ stress. Among the mutants, only *ΔFonPUF1* and *ΔFonPUF4* showed higher resistance to MgCl_2_ stress. Taken together, the variability in the sensitivity of the mutants to different stressors reveals that FonPUFs have critical but distinct roles in managing cell wall perturbing and abiotic stresses.

10.1128/mbio.00157-23.4 FIG S4 FonPUFs regulate the sensitivity to osmotic stress and ionic stress. (A) Growth phenotypes and (B) inhibition rates of the radial growth of WT and *ΔFonPUFs* grew on PDA containing NaCl, sorbitol, CaCl_2_, and MgCl_2_. Mycelial plugs of strains were inoculated on PDA supplemented with 0.7 M NaCl and 1 M sorbitol. Colony diameters were measured 7 days after incubation. Stress sensitivity was estimated by the growth inhibition rate (MGIR) using the formula MGIR% = [(N – C)/C] × 100, where C is the colony diameter grown on PDA and N is that with treatment. Each experiment was repeated three times independently. Data presented here are the means ±SD, and asterisks above the columns indicate the significant difference at *P < *0.05 level. Download FIG S4, EPS file, 7.6 MB. Copyright © 2023 Gao et al. 2023 Gao et al. https://creativecommons.org/licenses/by/4.0/ This content is distributed under the terms of the Creative Commons Attribution 4.0 International license.

### *FonPUF1*, but not other *FonPUFs*, is essential for *Fon* virulence on watermelon.

**FIG 3:**
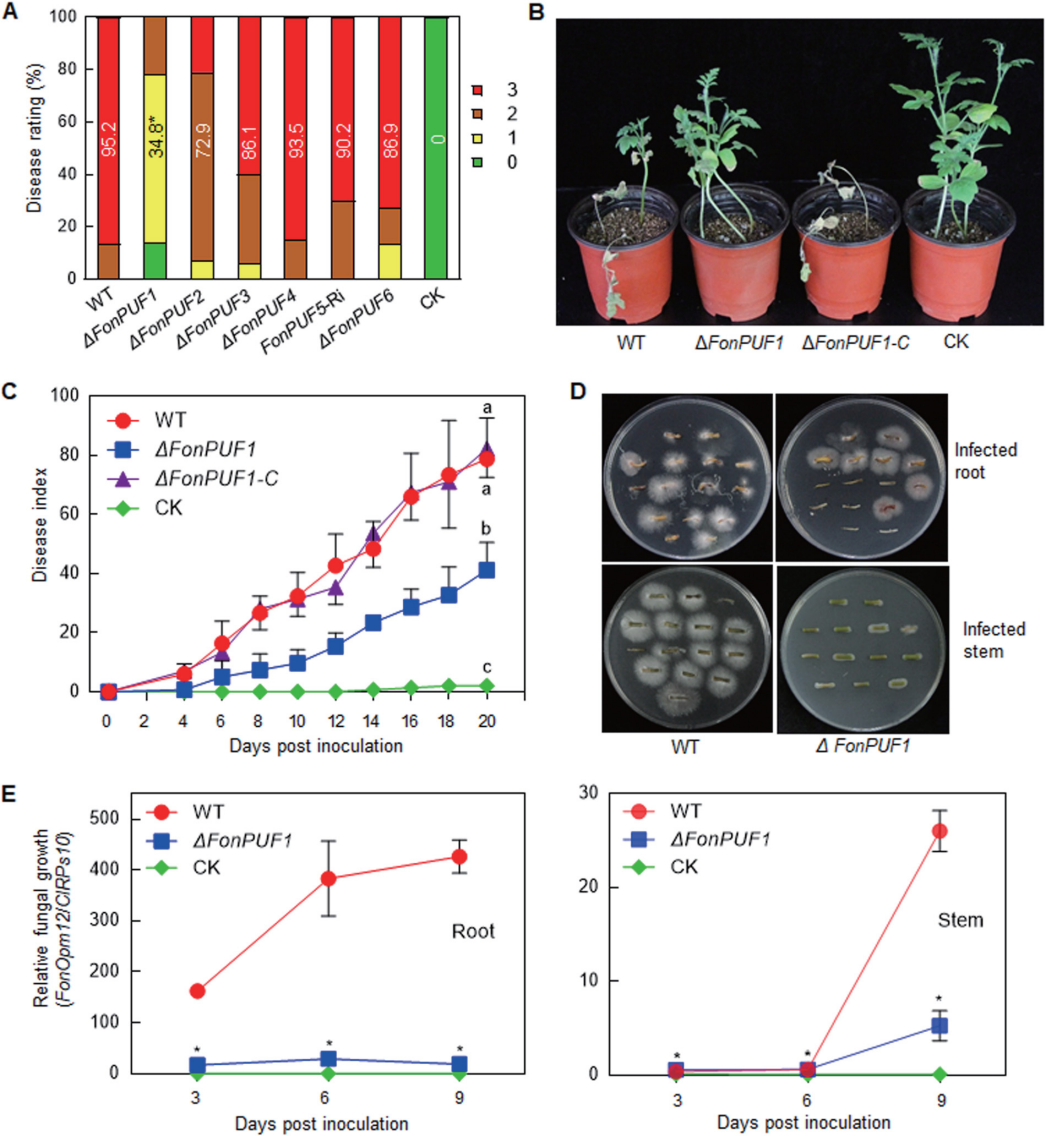
*FonPUF1* is required for the virulence of *Fon* on watermelon. (A) Disease ratings of watermelon plants inoculated by root dipping method with the WT and *ΔFonPUFs* deletion mutants at 3 weeks postinoculation. (B) Disease phenotype of watermelon plants inoculated with the WT, *ΔFonPUF1*, or *ΔFonPUF1*-C strains. Photographs were taken at 20 days postinoculation. (C) Disease index curve of watermelon plants inoculated with the WT, *ΔFonPUF1*, or *ΔFonPUF1*-C strains during a period of 20 days postinoculation. (D) Fungal colonies grown from the roots and stems of watermelon plants inoculated with the WT, *ΔFonPUF1*, or *ΔFonPUF1*-C strains. (E) Relative *in planta* fungal growth in the roots and stems of watermelon plants inoculated with the WT, *ΔFonPUF1*, or *ΔFonPUF1*-C strains. Relative fungal growth was quantified by qPCR analysis in terms of the transcript levels of *FonOpm12* or watermelon *ClRps10* and represented as the ratio of *FonOpm12*/*ClRps10*. Experiments in panels B and D were independently performed three times with similar results. Data presented in panels A, C, and E are the means ± SD from three independent experiments, and different letters in panel C above the error bars indicate statistically significant difference at *P < *0.05 level calculated by LSD test. Data in panels A and E annotated with asterisks are significantly different from the WT (*P < *0.05).

Disease assays were conducted to investigate whether *FonPUFs* have roles in *Fon* virulence on watermelon using the root infection method. Observations of disease phenotype and progress on the *ΔFonPUFs-* or *FonPUF5*-RNAi-inoculated plants revealed that the *ΔFonPUF1*-inoculated plants showed mild disease symptoms and a significantly lower disease severity index compared to WT-inoculated plants at 21 days postinoculation (Fig. 3A; see also Fig. S5). In contrast, the *ΔFonPUF2*-, *ΔFonPUF3*-, *ΔFonPUF4*-, *ΔFonPUF6*-, *FonPUF5*-RNAi-, and WT-inoculated plants exhibited comparable disease symptoms and progress (see Fig. S5). These results indicate that FonPUF1, but not other FonPFUs, is involved in *Fon* virulence on watermelon.

10.1128/mbio.00157-23.5 FIG S5 Disease phenotypes of watermelon plants inoculated with WT and *FonPUFs* knockout or knockdown strains. Disease phenotypes of inoculated watermelon plants by dipping root method. Disease phenotypes were photographed at 3 weeks postinoculation. Experiments were repeated at least three times with similar results, and results from one representative experiment are shown. Download FIG S5, EPS file, 7.1 MB. Copyright © 2023 Gao et al. 2023 Gao et al. https://creativecommons.org/licenses/by/4.0/ This content is distributed under the terms of the Creative Commons Attribution 4.0 International license.

To confirm whether the reduced virulence of *ΔFonPUF1* was due to the knockout of *FonPUF1*, a complementation strain *ΔFonPUF1*-C was generated by expressing the *FonPUF1* ORF in the respective mutant. Pathogenicity assays revealed that the *ΔFonPUF1*-C and WT-inoculated plants showed similar adverse disease symptoms and progress compared to the *ΔFonPUF1*-inoculated plants, exhibiting less severe disease symptoms and progress (Fig. 3B and C). These findings indicate that the knockout of *FonPUF1* reduced the virulence of *ΔFonPUF1* on watermelon. Fungal colonies recovered from *ΔFonPUF1*-infected root and stem tissues, especially from infected stem tissues, were apparently less than those from WT-inoculated plants (Fig. 3D). qPCR revealed that the fungal growth in *ΔFonPUF1*-infected root and stem tissues was significantly reduced by 96% and 82%, respectively, compared with that in WT-inoculated plants at 9 days postinoculation (Fig. 3E). As the penetration ability of *ΔFonPUF1* on cellophane membrane was not altered (see Fig. S6A), it is likely that the reduced virulence of *ΔFonPUF1* was due to defects in the invasive growth and colonization within the roots and stems of watermelon plants rather than the penetration ability. Collectively, these data indicate that FonPUF1 plays an important role in *Fon* virulence, mainly through regulating its invasive growth and colonization inside the watermelon plants.

10.1128/mbio.00157-23.6 FIG S6 Targeted disruption of *FonPUF1* or *FonARC18* does not affect the penetration ability and mitochondrial morphology and FonPUF1 does not alter the subcellular localization of FonARC18 in *Fon*. (A) Penetration ability of WT, *ΔFonPUF1*, *ΔFonARC18* strains against cellophane membranes. Mycelial plugs were placed on the prepared MM plates covered with cellophane membranes. The cellophane membranes along with the fungal colonies were removed after incubation for 3 days and the MM plates were incubated for another 2 days to observe the growth of mycelia. Experiments were repeated at least three times with similar results, and results from one representative experiment are shown. (B) Fluorescence signals (*top*) and linescan graphs (*bottom*) show the colocalization of fluorescence signals from FonPUF1-mcherry and Mito-Tracker. (C) Fluorescence signals and linescan graphs of FonARC18-mcherry and Mito-Tracker in WT background. (D) Fluorescence signals and linescan graphs of FonARC18-mcherry and Mito-Tracker in *FonPUF1* knockout background. White arrows in panels B to D indicated the areas used for linescan graph analysis. Scale bar = 5 μm. (D) Observation of mitochondrial morphology of WT, *ΔFonPUF1*, and *ΔFonARC18* strains using a transmission electron microscope. Each experiment was repeated three times independently with similar results. Download FIG S6, EPS file, 7.9 MB. Copyright © 2023 Gao et al. 2023 Gao et al. https://creativecommons.org/licenses/by/4.0/ This content is distributed under the terms of the Creative Commons Attribution 4.0 International license.

### RNA binding activity and the significance of the conserved domains in FonPUF1 biochemical activity and *Fon* virulence.

**FIG 4:**
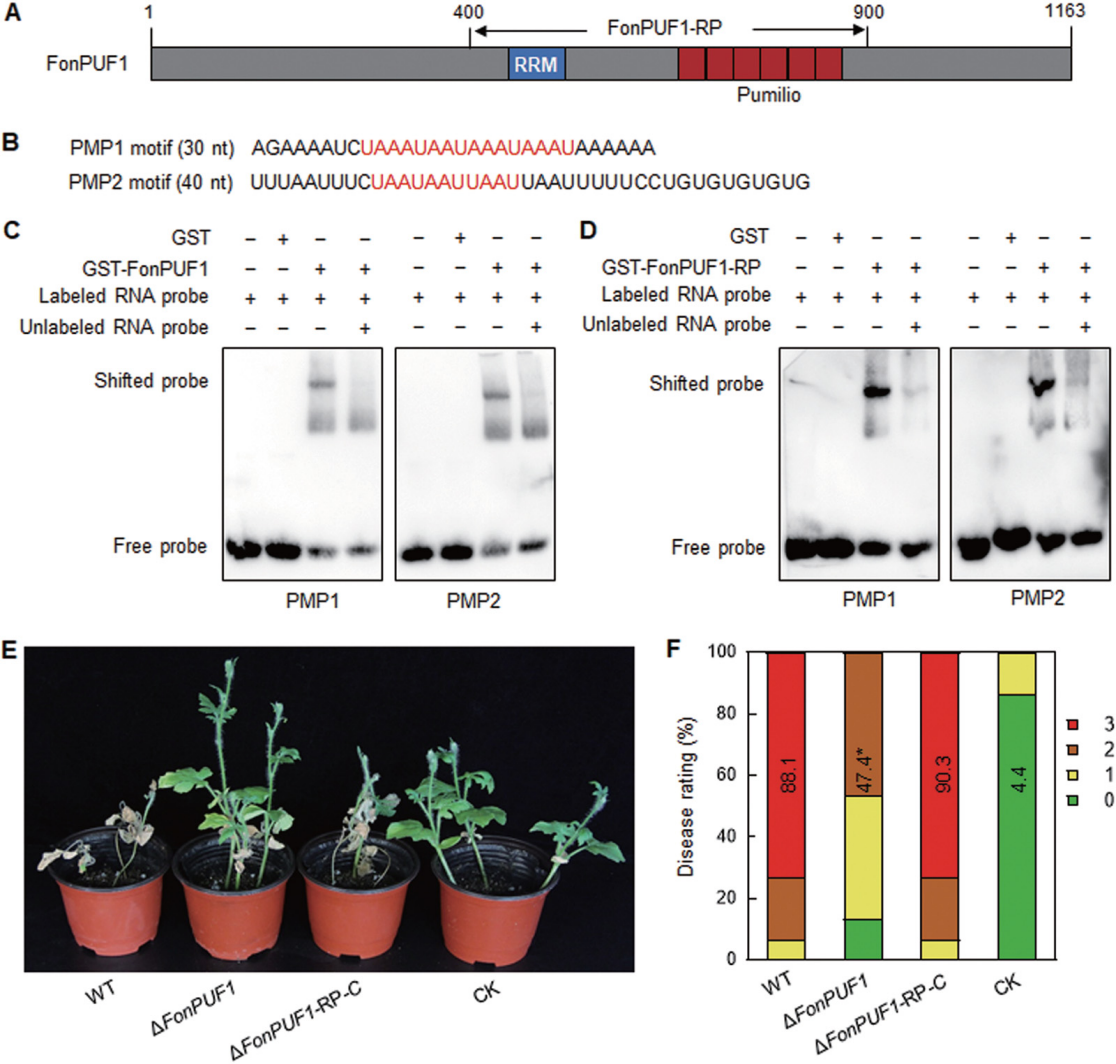
FonPUF1 binds to PMP1 and PMP2 *in vitro* and the RRM-Pumilio region is critical for FonPUF1 RNA binding activity and virulence of *Fon* on watermelon. (A) Schematic diagram of the domain organization of FonPUF1 and the FonPUF1-RP region consisting of RRM and Pumilio repeats. (B) Sequences of PMP1 and PMP2 used in the EMSAs. (C) Binding ability of FonPUF1 to PMP1 and PMP2 in the EMSAs. (D) Binding ability of FonPUF1-RP to PMP1 and PMP2 in the EMSAs. (E) Disease phenotype and (F) ratings of watermelon plants inoculated with the WT, *ΔFonPUF1*, and *ΔFonPUF1*-*RP*-C strains. Experiments in panels C, D, and E were independently performed three times with similar results. Data in panel F annotated with asterisks are significantly different from WT (*P < *0.05).

To explore the molecular mechanism of FonPUF1 in *Fon* virulence, its biochemical activity was investigated. As mentioned above, FonPUF1 contains a typical Pumilio domain with 6 Pumilio repeats and an additional RNA binding RRM domain (Fig. 4A; reference 24). Yeast ScPUF2 was previously shown to be capable of binding to PMP1 and PMP2 motifs (53). Considering the high structural and sequence similarity of FonPUF1 with ScPUF2 (see Fig. S1), the binding activity of FonPUF1 to PMP1 and PMP2 (Fig. 4B) was examined. In an electrophoretic mobility shift assay (EMSA), prokaryotically expressed and purified GST-FonPUF1 was found to bind with biotin-labeled PMP1 and PMP2, whose binding was significantly suppressed by excess unlabeled PMP1 and PMP2 (Fig. 4C). These results indicate a specific binding activity of FonPUF1 toward PMP1 and PMP2 motifs, implying a conserved biochemical activity of PUF proteins among different fungi. To explore the significance of the RRM and Pumilo domains in FonPUF1, a 500-amino-acid fragment of FonPUF1-RP harboring the RRM and Pumilio domains was generated (Fig. 4A). Similar to the full FonPUF1 fragment, prokaryotically expressed and purified GST-FonPUF1-RP showed binding activity toward biotin-labeled PMP1 and PMP2, which was significantly inhibited by excess unlabeled PMP1 and PMP2 (Fig. 4D). The *FonPUF1-RP* fragment was introduced into *ΔFonPUF1* to generate a complementation strain, *ΔFonPUF1-RP*-C. Pathogenicity tests indicated that the *ΔFonPUF1-RP*-C- and WT-inoculated plants had similar disease symptoms and severity index (Fig. 4E and F). These data suggest that RRM and Pumilio domains are critical for the biochemical activity and virulence function of FonPUF1.

### FonPUF1 interacts with the ARP2/3 complex.

**FIG 5:**
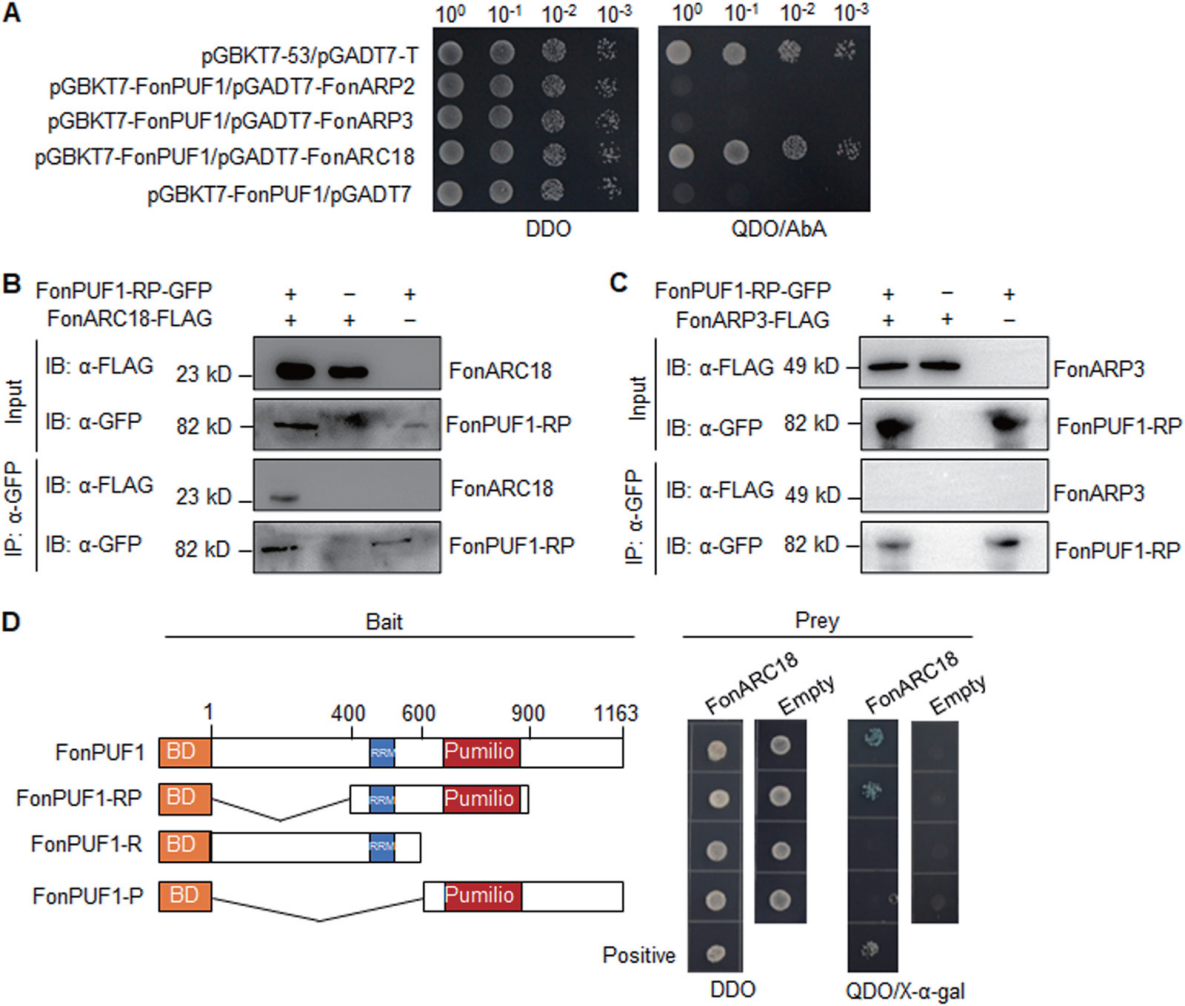
FonPUF1 interacts with mitochondria-associated FonARC18. (A) Interaction between FonPUF1 and the ARP2/3 complex components FonARC18, FonARP2, and FonARP3 in Y2H assay. (B) Interaction of FonPUF1-RP and FonARC18 in Co-IP assay. (C) Interaction of FonPUF1-RP and FonARP3 in Co-IP assay. Total proteins were extracted from *Fon* strains expressing FonPUF1-RP-GFP and FonARC18-FLAG or FonARP3-FLAG and IP was performed with anti-GFP agarose beads. IP samples were detected using anti-GFP and anti-FLAG antibodies, respectively. (D) The RRM and Pumilio domains in FonPUF1 are required for the FonPUF1-FonARC18 interaction in Y2H assay. Different truncated mutants of FonPUF1 were generated (*left*) and examined for their interaction ability with FonARC18 (*right*). Yeast cells cotransformed with indicated pairs of pGBKT7 and pGADT7 vectors were incubated on DDO (SD/-Trp/-Leu) and QDO (SD/-Ade/-His/-Leu/-Trp)+X-α-gal plates. Experiments were independently performed three times with similar results.

Yeast ScPUF1 (ScJsn1) was shown to physically interact with the mitochondria-associated Arp2/3 complex (45), which nucleates branched actin filament networks and regulates actin-based mitochondrial movement (54). To examine whether FonPUF1 associates with the FonArp2/3 complex, the interaction between FonPUF1 and the ARP2/3 complex components, including FonARP2, FonARP3, and FonARC18, was examined by yeast two-hybrid (Y2H) and coimmunoprecipitation (Co-IP) assays. In Y2H assays, FonPUF1 showed interaction with FonARC18, a small subunit of the FonArp2/3 complex but not with the FonARP2 and FonARP3 (Fig. 5A). Consistently, FonPUF1 was coimmunoprecipitated with FonARC18 but not with FonARP3 (Fig. 5B and C). These results suggest that FonPUF1 interacts with FonARC18 in the FonArp2/3 complex, which is in line with the previous observations in yeast (45). On the other hand, yeast ScPUF1 (ScJsn1) was shown to facilitate the association of the ARP2/3 complex to mitochondria (45). To determine the role of the conserved Pumilio and RRM domains in the FonPUF1-FonARC18 interaction, we created a series of truncated mutants of FonPUF1 (Fig. 5D). In Y2H assays, FonPUF1-RP showed interaction with FonARC18 (Fig. 5D). In contrast, FonARC18 interaction activity toward FonPUF1-R and FonPUF1-P was completely abolished (Fig. 5D). These findings suggest that both Pumilio and RRM domains play a constitutive role in FonPUF1 interaction with FonARC18. The subcellular localization of FonPUF1 and FonARC18 was further explored using WT expressing FonPUF1-mCherry and FonARC18-mCherry. Microscopic observations revealed that FonPUF1-mCherry and FonARC18-mCherry fusion proteins were colocalized in the mitochondria, as indicated by the mitochondrial marker Mito-Tracker Green (see Fig. S6B and C). Further, FonARC18-mcherry fusion protein expressed in *ΔFonPUF1* and WT was detected in the mitochondria (see Fig. S6D), indicating that the deletion of *FonPUF1* did not affect the subcellular localization of FonARC18. Collectively, these results suggest that FonPUF1 associates with the FonArp2/3 complex via interacting with its small subunit FonARC18 but does not affect the accumulation of the ARP2/3 complex in mitochondria.

### The FonARP2/3 complex component FonARC18 mediates *Fon* virulence.

**FIG 6:**
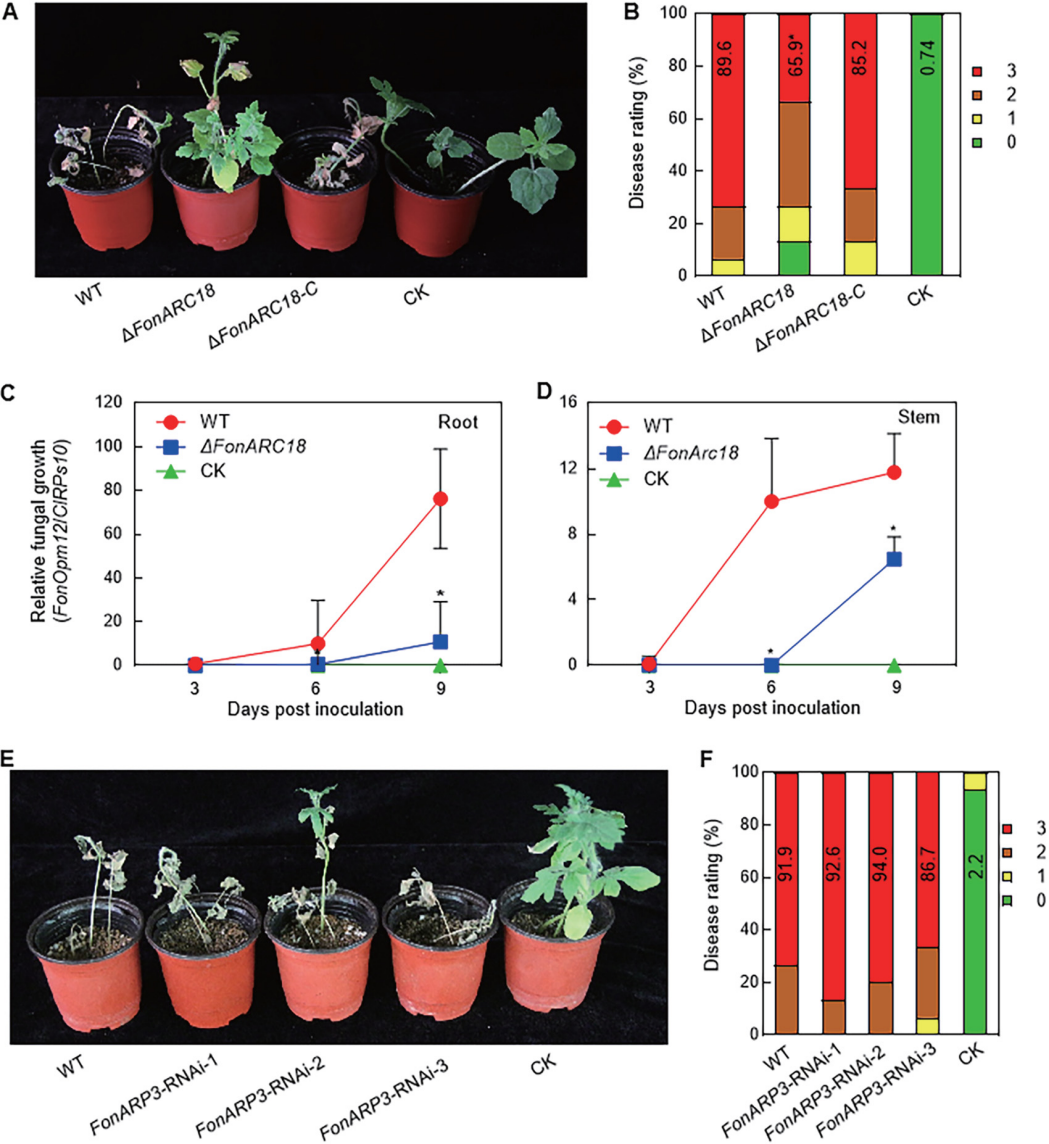
FonARC18, but not FonARP3, is required for the virulence of *Fon* on watermelon. (A and B) Disease phenotype (A) and ratings of watermelon plants (B) inoculated with WT, *ΔFonARC18*, and *ΔFonARC18*-C strains at 20 days postinoculation. (C and D) *In planta* fungal growth in the roots (C) and stems (D) of WT and *ΔFonARC18*-inoculated plants. Relative fungal growth was quantified by qPCR analysis in terms of the transcript levels of *FonOpm12* or watermelon *ClRps10* and represented as the ratio of *FonOpm12*/*ClRps10*. (E and F) Disease phenotype (E) and ratings of plants (F) inoculated with the WT and *FonARP3*-RNAi strains at 20 days postinoculation. Experiments in panels A and E were independently performed three times with similar results. Data presented in panels B, C, D, and F are the means ± SD from three independent experiments. Data in panels B, C, and D annotated with asterisks are significantly different from WT (*P < *0.05).

To examine whether the FonArp2/3 complex is involved in *Fon* virulence, the targeted deletion approach was used to generate the deletion mutant for *FonARC18*, which was further confirmed by Southern blotting (see Fig. S3D and E). Pathogenicity tests revealed that *ΔFonARC18* showed reduced virulence on watermelon, showing a 27% reduction in the disease index compared with WT (Fig. 6A). Approximately 60% of the *ΔFonARC18*-inoculated plants died, while 80% of the WT-inoculated plants died of wilting at 20 days postinoculation (Fig. 6B). Disease severity and index in the complementation strain *ΔFonARC18*-C- and WT-inoculated plants were similar to each other (Fig. 6A and B), indicating that the reduced virulence of *ΔFonARC18* was due to the deletion of *FonARC18*. qPCR quantification of *in planta* fungal biomass showed that the growth of *ΔFonARC18* in infected root (86%) and stem (48%) tissues was significantly reduced compared to WT-inoculated plants at 9 days postinoculation (Fig. 6C and D). However, the penetration ability of *ΔFonARC18* on the cellophane membrane remained intact (see Fig. S6A). These results indicate that FonARC18 regulates *Fon* virulence by regulating invasive growth and colonization inside the watermelon plants rather than the pathogen penetration ability.

Repeated attempts to generate deletion mutants for *FonARP2* and *FonARP3* failed, implying that the deletion of *FonARP2* or *FonARP3* might be lethal in *Fon*. This is consistent with similar observations in yeast, where the deletion of the ARP2/3 complex components resulted in lethality and severe reductions in viability (54). Therefore, RNAi-mediated knockdown *FonARP3* strains were generated using a pSilent1 vector with a 377-bp fragment in the *FonARP3* ORF as an RNAi target. RT-qPCR results showed that the transcript level of *FonARP3* in three *FonARP3*-RNAi strains was ~26%, ~32%, and ~65% of that in WT (see Fig. S3F). Pathogenicity tests revealed that disease severity and index in *FonARP3*-RNAi-inoculated plants were comparable to those in the WT-inoculated plants (Fig. 6E and F). These data indicate that FonARP3 is nonessential for *Fon* virulence.

### FonPUF1 and FonARC18 direct mitochondria-related functions.

**FIG 7:**
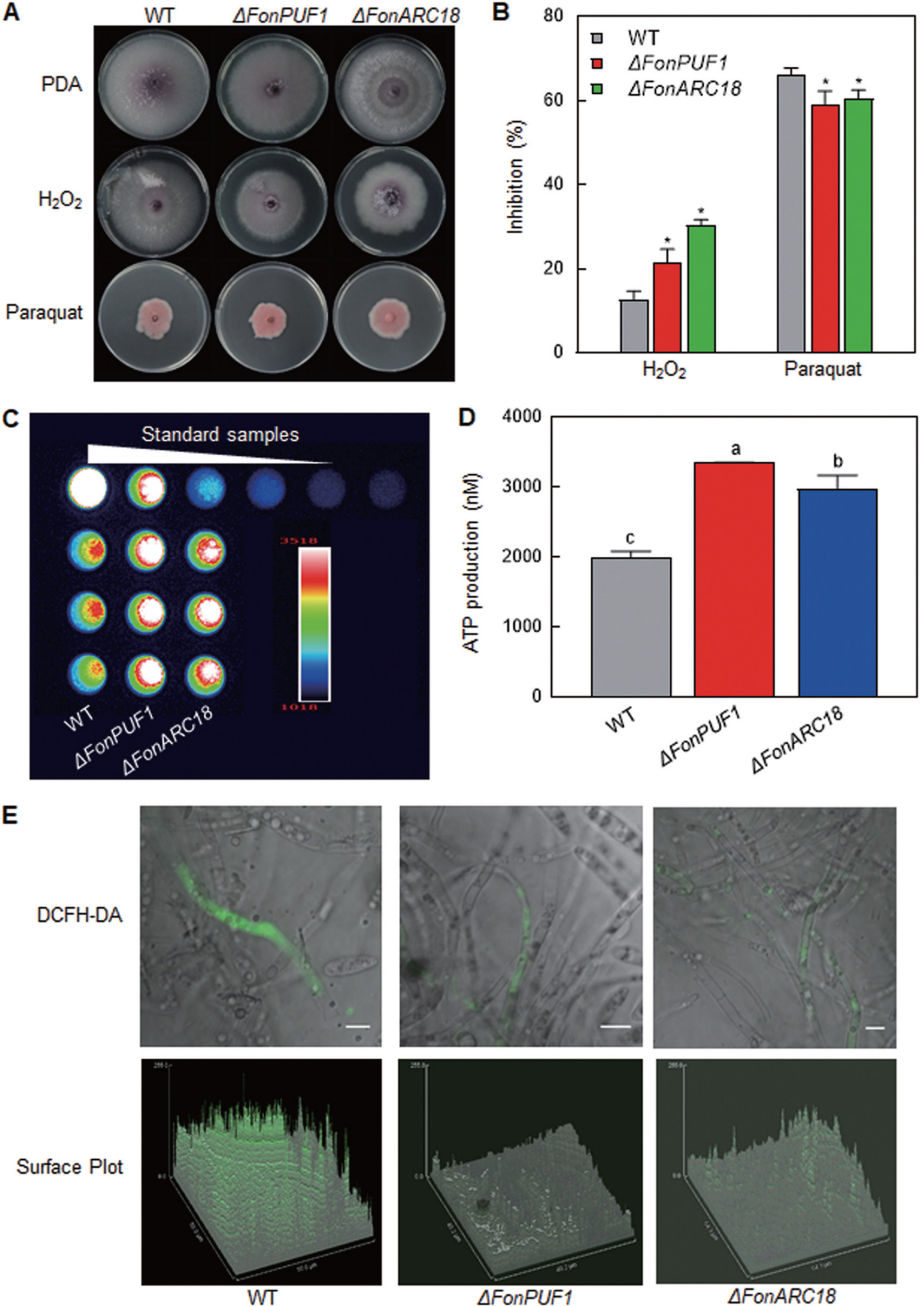
FonPUF1 and FonARC18 regulate mitochondrial functions. (A and B) Growth phenotype (A) and mycelial growth inhibition rate (B) of the WT, *ΔFonPUF1*, and *ΔFonARC18* strains grown on PDA with or without H_2_O_2_ or paraquat for 7 days. (C) ATP production in the WT, *ΔFonPUF1*, and *ΔFonARC18* strains. Luminescence from samples was measured in a luminometer. (D) ATP levels in the WT, *ΔFonPUF1*, and *ΔFonARC18* strains. (E) Reduced ROS in the *ΔFonPUF1* and *ΔFonARC18* strains. Hyphae grown in MM for 48 h were stained by ROS indicator DCFH-DA and surface plots were generated using the ImageJ software. Scale bar = 5 μm. Experiments in panels A, C, and E were independently performed three times with similar results. Data presented in panels B and D are the means ± SD from three independent experiments. Data in panel A annotated with asterisks are significantly different from WT (*P < *0.05), while different letters in panel D above the error bars indicate statistically significant difference at *P < *0.05 level calculated by LSD test.

In yeast, ScPUF1 (ScJsn1) and the ScArp2/3 complex play important roles in modulating mitochondrial functions (45). The localization of FonPUF1 and FonARC18 in mitochondria (see Fig. S6B to D) suggests that these two proteins can be involved in controlling mitochondrial activities. Transmission electron microscopy (TEM) observations indicated that the morphology and structure of mitochondria in *ΔFonPUF1* and *ΔFonARC18* were similar to those in WT (see Fig. S6E). It is well-known that oxidative stress and mitochondrial dysfunction are generally correlated (55). The sensitivity of *ΔFonPUF1* and *ΔFonARC18* to oxidative stress was examined and compared with WT. When grown on PDA supplemented with 5 mM hydrogen peroxide (H_2_O_2_) and 3 mM paraquat, *ΔFonPUF1* and *ΔFonARC18* exhibited significantly increased sensitivity to H_2_O_2_, while they showed higher tolerance to paraquat, compared to WT (Fig. 7A and B). In eukaryotic cells, mitochondria play a key role in the production of endogenous reactive oxygen species (ROS) and ATP (56). *ΔFonPUF1* and *ΔFonARC1* produced higher levels of ATP, resulting in an increase of 68% and 50%, respectively, compared with those in WT (Fig. 7C and D). The 2′,7′-dichlorofluorescein diacetate (DCFH-DA) staining, fluorescence observation, and surface plot analyses showed that the hyphae of *ΔFonPUF1* and *ΔFonARC18* produced less ROS than WT (Fig. 7E). The aberrant levels of ATP and ROS reflect the mitochondrial dysfunction in *ΔFonPUF1* and *ΔFonARC18*. Collectively, these results suggest that FonPUF1 and FonARC18 play important roles in regulating mitochondrial functions, such as ATP generation and ROS production.

### Identification of FonPUF1-affected genes.

**FIG 8:**
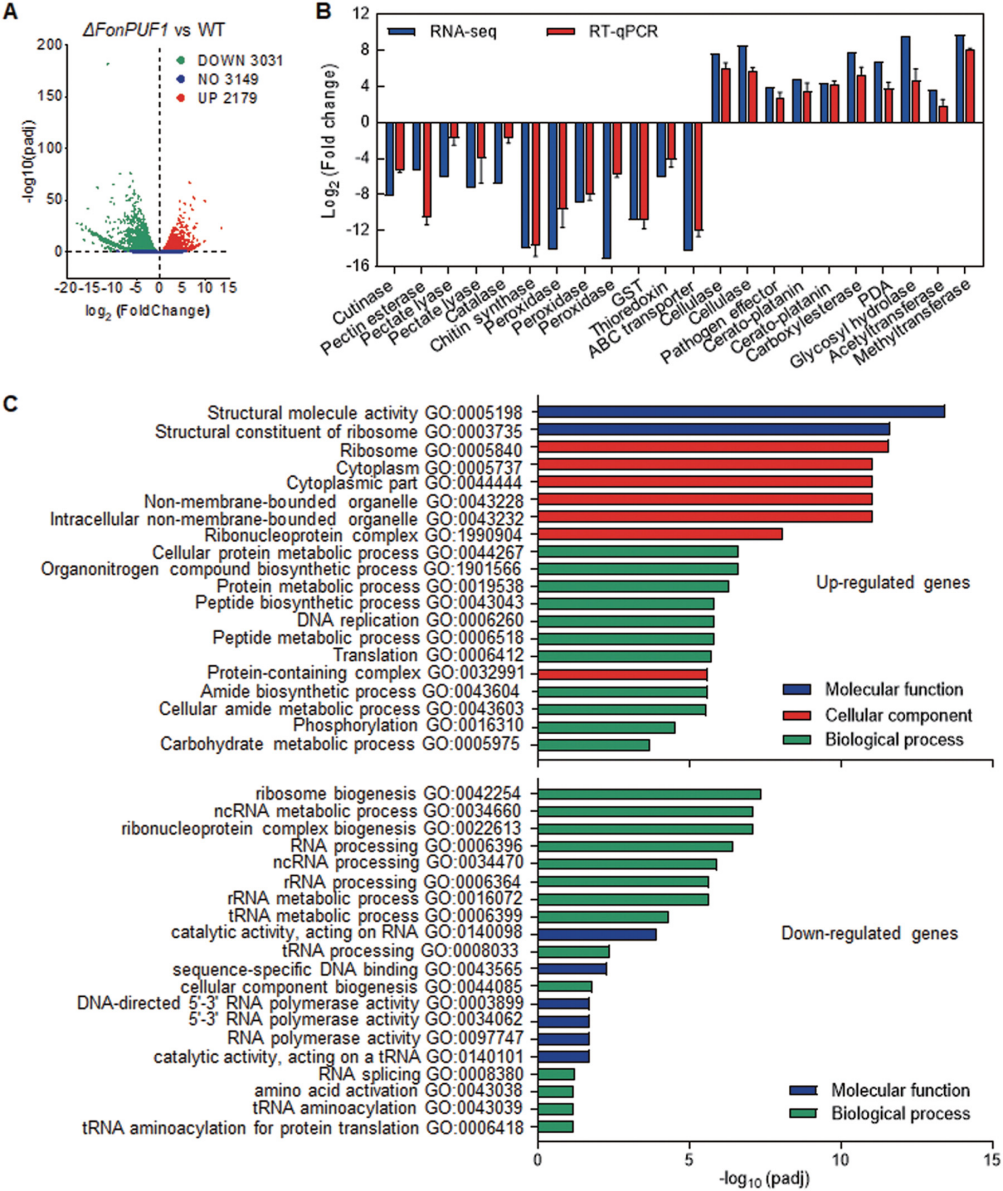
Analysis of the FonPUF1-affected transcripts in *Fon*. (A) A volcano plot of the upregulated or downregulated DEGs in *ΔFonPUF1*. (B) RT-qPCR validation of the expression changes of 10 upregulated and 12 downregulated DEGs selected from RNA-seq data. (C) GO enrichment analyses of upregulated and downregulated DEGs from RNA-seq data. Main GO categories include biological processes, cellular component, and molecular function for upregulated DEGs, while biological processes and molecular functions for downregulated DEGs. Three independent biological replicates from *ΔFonPUF1* and WT were used for transcriptome analysis.

To explore the underlying molecular regulatory mechanism of FonPUF1 in *Fon* virulence, RNA-sequencing-based transcriptome profiling (RNA-seq) was performed to identify a cascade of FonPUF1-regulated genes in *Fon*. A total of 54,000,000 to 67,000,000 reads were obtained from 6 samples (3 *ΔFonPUF1* and 3 WT) and mapped to the F. oxysporum f. sp. *lycopersici* reference genome. The ratio of RNA-seq reads mapped to the F. oxysporum genome ranged from 61% to 80%, and >79% of the reads were annotated to exon regions (see Fig. S7). In contrast to WT, a total of 5,210 genes were characterized as DEGs in *ΔFonPUF1*, exhibiting 3,031 downregulated DEGs and 2,179 upregulated DEGs (Fig. 8A). Interestingly, some putative virulence-related genes were differentially expressed in *ΔFonPUF1* (see Table S2). To validate the RNA-seq results, the expression changes of 10 upregulated and 12 downregulated DEGs were confirmed by RT-qPCR and found comparable to those in RNA-seq analysis (Fig. 8B). Genes encoding cell wall-degrading enzymes, including cutinase, pectinesterase and pectate lyase (8, 57), enzymatic antioxidants, including peroxidase and catalase (58), and cell wall integrity/pathogenicity-related chitin synthase (59), were significantly downregulated in *ΔFonPUF1*. In contrast, genes for host defense activating effectors and cerato-platanin proteins (60) were markedly upregulated in *ΔFonPUF1*. The expression of some putative virulence-related genes, such as cellulase, ABC transporter, polysaccharide deacetylase, and thioredoxin (61–64), was also affected in *ΔFonPUF1*. Therefore, it is likely that FonPUF1 regulates the expression of a set of putative virulence-related genes in *Fon*.

10.1128/mbio.00157-23.7 FIG S7 Proportion of the sequencing reads mapped to the genome in the RNA-seq analysis. Download FIG S7, EPS file, 7.5 MB. Copyright © 2023 Gao et al. 2023 Gao et al. https://creativecommons.org/licenses/by/4.0/ This content is distributed under the terms of the Creative Commons Attribution 4.0 International license.

10.1128/mbio.00157-23.9 TABLE S2 Putative virulence-related genes regulated by FonPUF1. Download Table S2, XLSX file, 0.01 MB. Copyright © 2023 Gao et al. 2023 Gao et al. https://creativecommons.org/licenses/by/4.0/ This content is distributed under the terms of the Creative Commons Attribution 4.0 International license.

Gene ontology (GO) enrichment analysis categorized up/downregulated DEGs into 20 GO terms (*P* adjusted <0.05) (Fig. 8C). The significantly enriched GO terms include ribosome biogenesis, ribonucleoprotein complex biogenesis, rRNA processing, rRNA metabolic process, and organonitrogen compound biosynthetic process, indicating that FonPUF1 affects several physiological processes associated with ribosome- and rRNA-related metabolic pathway. Similar findings were reported in yeast, demonstrating that ScPuf1 and ScPuf2 altered the expression of a set of genes encoding ribosome-associated and rRNA metabolism-related proteins (44).

### Identification of a new binding motif for FonPUF1.

**FIG 9:**
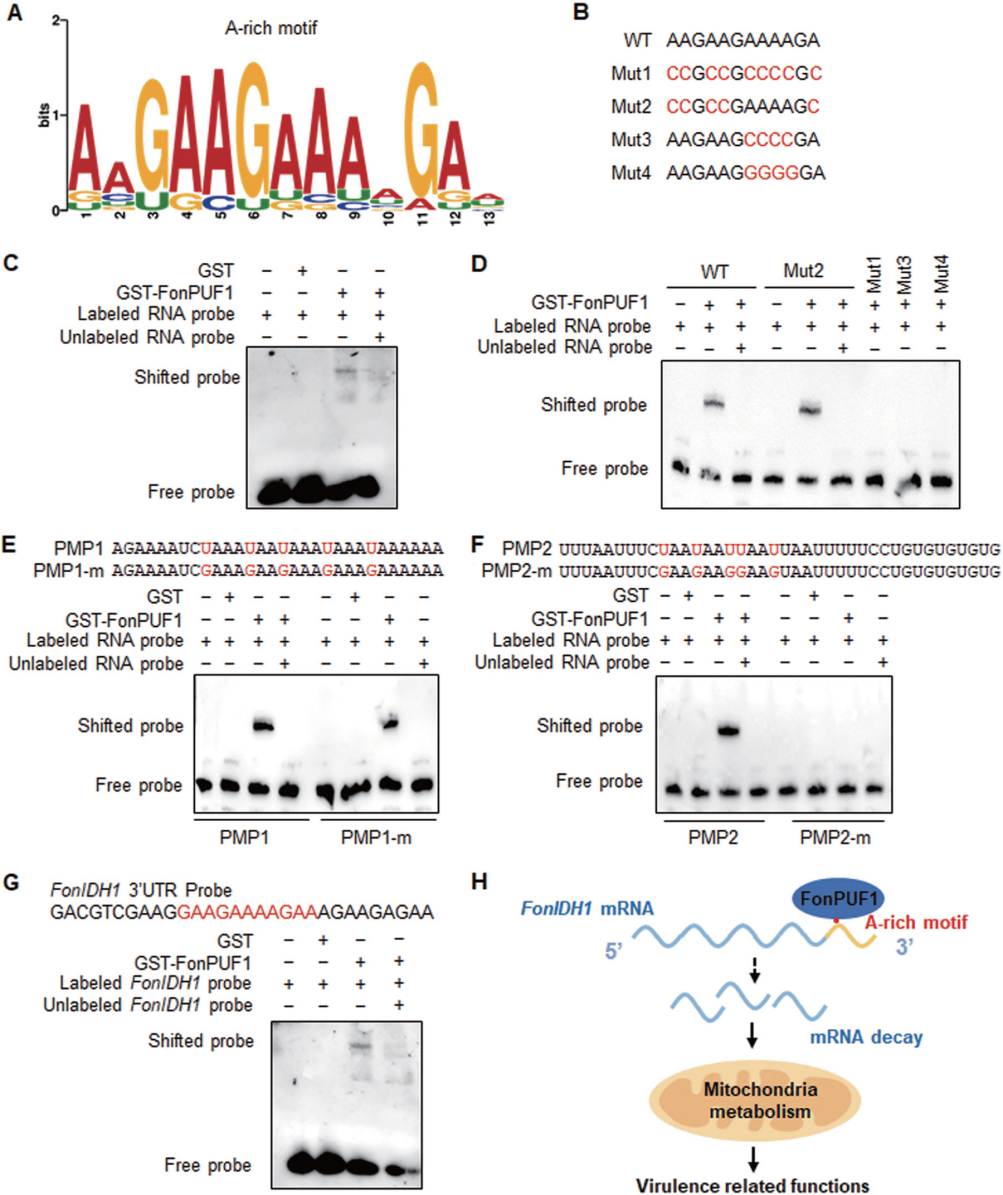
FonPUF1 binds to an A-rich motif in 3′ UTR of target mRNA. (A) Consensus enriched motif sequence in the 3′ UTR of the upregulated DEGs in *ΔFonPUF1.* The *y* axis (*letter size*) indicates the information content (*bits*) of each position. The motif search was performed using the MEME program (https://meme-suite.org/meme/tools/meme). (B) Sequences of WT and mutated A-rich motif. The mutated sites in modified probes are indicated in red. (C) Binding ability of FonPUF1 to A-rich motif. (D) Binding ability of FonPUF1 to mutated versions of the A-rich motif. (E) Binding ability of FonPUF1 to WT and mutated PMP1 motif. (F) Binding ability of FonPUF1 to WT and mutated PMP2 motif. The mutated sites in modified probes are indicated in red (E and F). (G) EMSA analysis of FonPUF1 using *FonIDH1* 3′-UTR probe. Biotin-labeled RNA probes were incubated with purified GST-FonPUF1 or GST for 30 min at 28°C. Experiments were independently performed three times with similar results, and results from one representative experiment are shown. (H) A working model of FonPUF1 deciphering its roles in regulating mitochondria-related functions and *Fon* virulence via binding to the 3′-UTR region of *FonIDH1*.

Mounting evidence suggests that PUF proteins mainly function as transcriptional repressors through destabilizing and degrading target mRNAs by binding at 3′ UTR (65). To identify new putative binding motifs of FonPUF1, the 200-bp 3′-UTR sequences of 949 significantly upregulated DEGs in *ΔFonPUF1* were analyzed using the Multiple EM for Motif Elicitation (MEME) suite. The MEME analysis identified a conserved A-rich motif in the 3′-UTR sequences of the upregulated DEGs (Fig. 9A). However, MEME analysis of the 3′-UTR sequences of the downregulated DEGs in *ΔFonPUF1* did not discover any conserved motif. To verify the binding capacity of FonPUF1 to this newly identified A-rich motif, biotin-labeled A-rich motif and three mutated versions were created (Fig. 9B). In the EMSAs, GST-FonPUF1 showed binding activity toward biotin-labeled A-rich motif, as revealed by the appearance of a shifted band above the free probe, and the binding of GST-FonPUF1 to this A-rich motif was suppressed by the excess unlabeled probe (Fig. 9C). GST-FonPUF1 did not show binding activity toward the mut1, mut3, and mut4 RNA sequences (Fig. 9D), containing mutated As at the 7th, 8th, 9th, and 10th positions (Fig. 9B); however, GST-FonPUF1 was found to bind with the mut2 mRNA sequence (Fig. 9D), comprising mutated As at the 1st, 2nd, 4th, and 5th positions, while the 7th, 8th, 9th, and 10th positions were intact (Fig. 9B). These data demonstrate that the A-rich motif is a novel binding motif for FonPUF1 and As at the 7th, 8th, 9th, and 10th positions are critical for its binding activity with FonPUF1. Further, we noticed certain sequence similarity among the A-rich motif the PMP1 or PMP2 motifs. Thus, we mutated U to G in primary sequences of PMP1 and PMP2. Shifted bands were appeared with the mutated PMP1 probe (Fig. 9E). However, GST-FonPUF1 was incapable to bind and shift the mutated PMP2 probe (Fig. 9F). It follows that the A-rich motif might represent a different isoform of PMP1.

### FonPUF1 binds to the A-rich motif in its target *FonIDH1* mRNA.

To further investigate the biochemical activity of FonPUF1, we examined FonPUF1 binding to the A-rich motif in its target mRNAs. A MEME search identified another putative binding target sequence (AAGAAGAAAAGA) for FonPUF1. From MEME results, we found 11 genes, including isocitrate dehydrogenase (IDH)-encoding gene *FonIDH1*, containing the AAGAAGAAAAGA motif at their 3′ UTR. IDH, a key tricarboxylic acid (TCA) cycle component (66), produces ATP in mitochondria via controlling the TCA cycle. Given these facts, IDH dysfunction can disrupt the TCA cycle and lead to a severe energy deficit as well as impairments in the ROS-balancing system (67). To further validate the pivotal role of FonPUF1 in mitochondrial functions, we investigated the binding activity of FonPUF1 with *FonIDH1* mRNA. The EMSAs were conducted using *FonIDH1* mRNA, which contained a synthetic RNA probe with an AAGAAGAAAAGA motif in its 3′-UTR region. GST-FonPUF1 bound to the modified *FonIDH1* containing the 3′-UTR probe and retarded its migration (Fig. 9G). Moreover, transcriptomic data revealed that *FonIDH1* was upregulated in *ΔFonPUF1.* These facts suggest that *FonIDH1* can be a putative target of FonPUF1. Unfortunately, however, we failed to obtain the deletion mutant for *FonIDH1* in several independent attempts and thus were unable to investigate the biological functions of *FonIDH1* in *Fon*.

## DISCUSSION

PUFs, a class of highly conserved RNA binding proteins, play essential roles in the posttranscriptional regulation of gene expression in higher eukaryotes (23, 68–70). However, their role in plant pathogenic fungi is still elusive. The present study revealed that *FonPUF1*, but not other *FonPUFs*, is required for *Fon* virulence on watermelon (Fig. 3). F. oxysporum has evolved a unique infection behavior toward host plants, where the hyphal attachment to the root surface, followed by the penetration, entry, and *in planta* colonization within the vascular system are important for full virulence on the host plants (71). The disruption of *FonPUF1* did not affect the penetration ability of *ΔFonPUF1* on cellophane membrane (see Fig. S6A); however, the fungal recovery rate and *in planta* growth of *ΔFonPUF1* in the roots and stems of infected watermelon plants were dramatically reduced (Fig. 3D and E), suggesting an attenuated ability of *ΔFonPUF1* to invade and colonize inside the watermelon plants. This is consistent with previous findings in *Fon*, in which the disruption of *FonNst2*, a nucleotide sugar transporter, reduced *Fon* virulence on watermelon by affecting *in planta* fungal colonization rather than the pathogen penetration ability (52). These facts suggest that FonPUF1 plays a critical role in *Fon* virulence by regulating the invasive growth and colonization ability inside the watermelon plants, thus providing new opportunities to unravel the underlying molecular mechanism of Pumilio proteins linked with the virulence in *Fon*.

Similar to yeast, the *Fon* genome harbors six *FonPUF* genes. However, the FonPUF family exhibited an unorthodox relationship with yeast ScPUFs (see Fig. S1B). The underlying fact explaining the unconventional relationship of FonPUFs is that two yeast PUFs, ScJsn1 and ScPUF2, contain the RRM domain (29, 72), while only FonPUF1 harbors the RRM domain in *Fon* (see Fig. S1A), which is similar to M. oryzae and F. graminearum. PUF proteins are highly conserved in terms of protein structure, including domain sequence and organization. Notably, PUF proteins, such as ScJsn1 and ScPUF2 (23), typically contain 8 Pumilio repeats (73). Conversely, FonPUF3-6 have less than 8 Pumilio repeats (see Fig. S1A). The structural variations in the PUF family might be due to their evolutionary and functional divergence across different species. It was discovered that PUF proteins bind to conserved RNA motifs, such as PMP1 and PMP2 identified for the yeast ScPUFs (41). The present study revealed that FonPUF1 bound to PMP1 and PMP2 motifs *in vitro* (Fig. 4). In addition to previously reported motifs (41, 74), a novel A-rich motif, AAGAAGAAAAGA, was identified through MEME analysis of the 200 bp in the 3′ UTR of the upregulated DEGs in *ΔFonPUF1*. Intriguingly, FonPUF1 showed *in vitro* binding activity to the identified A-rich motif (Fig. 9). Mutation analysis revealed that the As at the 7th, 8th, 9th, and 10th positions are critical for FonPUF1 binding with its target mRNA (Fig. 9). Thus, it appears that in addition to species-specific RNA motif binding, FonPUF1 can also bind to general RNA motifs, implying its conserved role in fungi. In general, the RRM domain is responsible for binding to the target single-stranded RNAs (21), while the Pumilio domain physically binds to the 3′ UTR of the target mRNA (75). In the present study, we discovered that the FonPUF1-RP, which contained the RRM and Pumilio domains, exhibited a similar *in vitro* binding activity to PMP1 and PMP2 motifs as the full FonPUF1 (Fig. 4). Similarly, it was previously shown that the Pumilio domain of PuM90 bound to a motif in the mRNA 3′ UTR of a target gene *PuFLP* in the oomycete Pythium ultimum (76). Intriguingly, the FonPUF1-RP region partially restored the virulence defect in the *ΔFonPUF1* strain (Fig. 4). Overall, this set of evidence highlights the importance of the RRM and Pumilio domains for the biochemical activity and biological functions of FonPUF1; however, the significance of FonPUF1 N and C-terminals in regulating its functions in *Fon* needs to be investigated.

PUF proteins have been implicated in fungal growth and development. For example, it has been demonstrated that Aspergillus nidulans PufA and PufE are necessary for growth, sexual development, and spore viability (77). A variety of phenotypic analyses revealed that FonPUFs play differential roles in mycelial growth, macroconidia morphology or asexual reproduction, and spore germination in *Fon* (Fig. 1). Specifically, the disruption of *FonPUF1* led to significant growth and developmental defects (Fig. 1), indicating the importance of *FonPUF1* in regulating the biology of *Fon*. The roles of PUFs in managing cell wall and abiotic stresses have also been established in fungi. For example, yeast ScJsn1, ScPUF2, ScPUF3, and ScPUF5 play important roles in the regulation of temperature sensitivity, high Ca^2+^ and oxidative stress, and cell wall integrity (44, 78, 79), while S. pombe SpPUF2 is involved in the formation of stress granules and processing bodies (31). In the present study, FonPUFs display variable roles in the regulation of responses to the cell wall, metal ions, and osmotic stressors (Fig. 2; see also Fig. S4). Precisely, the disruption of *FonPUF1* resulted in an enhanced sensitivity to CR, CFW, NaCl, and sorbitol but a decreased sensitivity to SDS and MgCl_2_. Overall, FonPUFs differentially regulate multiple detoxification processes to counter abiotic stresses in *Fon*.

Interaction studies revealed that FonPUF1 interacted with the ARP2/3 complex via FonARC18 but not FonARP3 (Fig. 5). The disruption of *FonARC18* resulted in significantly reduced *Fon* virulence; however, the knockdown of *FonARP3* did not affect *Fon* virulence (Fig. 6). These findings suggest that FonARC18, but not FonARP3, is a crucial component of the ARP2/3 complex that regulates *Fon* virulence via interacting with FonPUF1, which is consistent with the previous observations that highlighted the importance of ARC18 as a key component of the ARP2/3 complex in regulating its function (49, 80). In yeast, ScPUF1/ScJsn1 has been shown to facilitate the association of Arp2/3 complex to mitochondria, thereby regulating mitochondrial morphology and functions (45). The disruption of genes encoding mitochondrial-localized FonPUF1 and FonARC18 did not alter the mitochondrial morphology (Fig. 5 and see Fig. S6E). However, a series of mitochondrial-related metabolic processes, including oxidative stress response, ATP production, and ROS production, were affected in *ΔFonPUF1* and *ΔFonARC18* (Fig. 7). It was previously found that mitochondrial integrity and ROS homeostasis are necessary for the virulence of *Fon* on watermelon, F. graminearum on wheat, and M. oryzae on rice (51, 81–83). Overall, these findings imply that FonPUF1 and the FonARP2/3 complex constitutively contribute to the mitochondrial functions via interacting with each other through FonARC18 and thus play a critical role in *Fon* virulence.

The posttranscriptional regulation of mRNA abundance is critical to the gene functions and thus plays an important role in growth and development of eukaryotes. PUFs, a group of RNA-binding proteins, control the stability, translation, and localization of the target mRNAs (23, 84). The abundance of target mRNA is directly linked to the PUF protein binding to its 3′ UTR that destabilizes the target RNA (23, 85–88). In this regard, an A-rich FonPUF1 binding motif was identified in the 3′ UTR of the upregulated DEGs, but not in the downregulated DEGs, in *ΔFonPUF1* (Fig. 9), which corroborated that mRNAs of the upregulated DEGs in *ΔFonPUF1* might be the targets of FonPUF1. Thousands of up/downregulated DEGs were identified in *ΔFonPUF1* by transcriptome analysis, and GO enrichment terms indicated that DEGs belonged to diverse biochemical pathways, including the ribosome-related or rRNA metabolic pathway (Fig. 8). This is consistent with the findings in yeast deciphering that genes related to ribosome biogenesis and rRNA processing were significantly enriched in *ΔScPUF1* strain (44). Therefore, it is likely that FonPUF1 and ScPUF1 (ScJsn1), which are phylogenetically linked and have similar protein structures and domain organizations (see Fig. S1), share common biological functions. In the present study, RNA-seq analyses identified a number of virulence-related DEGs in *ΔFonPUF1* (Fig. 8B). A set of secreted proteins known as Cerato-Platanin proteins from filamentous fungi has been reported to activate the defense response in host plants (89, 90); for example, FocCP1 from F. oxysporum f. sp. *cubense* triggered the immune response in plants (60). Similarly, cellulase has been shown to elicit immune responses in host plants (61). The upregulated DEGs encoding putative Cerato-Platanin, cellulases, and effectors in *ΔFonPUF1* (Fig. 8B) might be involved in the activation of the immune response in *ΔFonPUF1*-inoculated plants, thus providing a plausible explanation for the attenuated virulence of *ΔFonPUF1*. Conversely, cutinase and thioredoxin in *M. grisea* regulated appressorium differentiation and penetration ability toward rice plants (62, 91). Similarly, pectin-degrading enzymes were shown to weaken the host cell wall and thus play a critical role in the infection process of various phytopathogenic fungi (92). Mutations in the chitin synthase genes led to reduced pathogenicity of F. oxysporum on tomato (59), while an ABC transporter, FgArb1, was also found necessary for penetration of F. graminearum (63). In the present study, genes encoding cutinase, pectate lyases, chitin synthases, and an ABC transporter were found to be significantly downregulated in *ΔFonPUF1* (Fig. 8), indicating that FonPUF1 affects the abundance of these virulence-related genes in *Fon*. Therefore, it is likely that FonPUF1 regulates *Fon* virulence by destabilizing mRNAs of host defense-eliciting genes through binding to the A-rich motif in their 3′ UTR and/or affects the expression of a set of virulence-related genes through an unknown mechanism. Moreover, we identified *FonIDH1*, a key TCA cycle regulator, as a putative target of FonPUF1 through transcriptomic and biochemical analyses. Thus, it is likely that FonPUF1 regulates mitochondrial metabolism and *Fon* virulence via controlling the mRNA abundance of *FonIDH1*. However, the molecular mechanisms by which FonPUF1 controls the expression of *FonIDH1* to regulate mitochondrial functions and *Fon* virulence needs to be investigated.

In conclusion, the present study revealed that FonPUF1 regulates *Fon* virulence by affecting the invasive growth and colonization ability inside the watermelon plants. FonPUF1 and the FonARP2/3 complex collectively regulate mitochondrial functions via interacting through FonARC18. Moreover, FonPUF1 binds to an A-rich motif present in the 3′ UTR of target mRNAs and controls the abundance of a diverse set of genes, including virulence-related genes. Transcriptomic analysis identified *FonIDH1*, a key TCA cycle component, as a putative target for FonPUF1, which was further confirmed through biochemical analyses. The identification of this novel FonPUF1 target will enable us to further characterize and explore the virulence-related molecular mechanism in *Fon*. Based on these findings, a working model is proposed explaining the molecular mechanism by which FonPUF1 regulates virulence-related functions in *Fon* (Fig. 9H). Collectively, the advanced understanding regarding how FonPUF1 or its targets regulate different biological processes including virulence in *Fon* will help to establish a sustainable disease management strategy against phytopathogenic fungi.

## MATERIALS AND METHODS

### Fungal strains and culture conditions.

*Fon* race 1 strain ZJ1 was used as the WT strain. For vegetative growth assays, *Fon* strains were cultivated on PDA (200 g potato, 20 g glucose, and 10 g agar L^−1^ ddH_2_O) or MM [10 mM glucose, 10 mM KH_2_PO_4_,10 mM K_2_HPO_4_, 0.45 mM CaCl_2_, 4 mM (NH_4_)_2_SO_4_, 2.5 mM NaCl, 2 mM MgSO_4_, 9 mM FeSO_4_, and 10 g agar L^−1^ ddH_2_O, pH 6.9] and allowed to grow at 26°C. For conidiation assays, MBL broth (20 g mung beans L^−1^ ddH_2_O boiled for at least 20 min) was used. For spore germination, macroconidia were added into yeast extract peptone dextrose broth (YEPD; 3 g yeast extract, 10 g peptone, and 20 g dextrose L^−1^ ddH_2_O, pH 7.0) and incubated at 26°C in a rotary shaker at 200 rpm. For stress response assays, mycelial plugs were inoculated on PDA plates supplemented with different reagents and incubated at 26°C for 7 days. Cell wall stress was applied by adding 0.02% CR (Sigma-Aldrich, St. Louis, MO, USA), 0.02% CFW (Sigma-Aldrich, St. Louis, MO, USA), and 0.03% SDS (Sigma-Aldrich, St. Louis, MO, USA), while oxidative stress was induced by supplementing PDA with 5 mM H_2_O_2_ or 3 mM paraquat. For osmotic and ionic stress, PDA was supplemented with 0.7 M NaCl, 1 M sorbitol, 0.5 M CaCl_2_, or 0.1 M MgCl_2_. Stress sensitivity was calculated by the mycelial growth inhibition rate (MGIR) using the formula MGIR% = [(N − C)/C] × 100, where C is the diameter of colonies grown without stress and N is the diameter of colonies grown with a particular stressor (82).

### Generation of deletion, knockdown, and complementation strains.

The double-joint PCR method (93) was used to generate deletion mutants for *FonPUF* genes. A cassette containing the 1349 bp *HPH* fragment, the 5′- and 3′-flanking sequences of the target gene was constructed and transformed into the WT protoplasts. The primers used to amplify the 5′- and 3′-flanking sequences of the genes studied and *HPH* fragment are listed in Table S3. Fresh protoplasts were prepared using the polyethylene glycol (PEG) method (94). Briefly, fresh mycelia were lysed for 3 h at 30°C using a lysis enzyme cocktail, containing driselase (Sigma-Aldrich, St. Louis, MO, USA), lysozyme (RYON, Shanghai, China), and cellulose (RYON, Shanghai, China), and the released protoplasts were collected by centrifugation at 4,000 rpm and 4°C. After transformation by mixing the knockout fragments with protoplasts at room temperature for 20 min, putative transformants were selected on PDA supplemented with 100 μg mL^−1^ hygromycin B (Hyg) and confirmed through PCR using gene-specific primers (see Table S3). To further validate the deletion of the target genes, Southern blotting was performed using a previously described protocol (51). Briefly, genomic DNA was digested with specific restriction enzymes, separated by electrophoresis on the agarose gel, and transferred to a nylon membrane (Millipore, Billerica, MA, USA). Prehybridization, hybridization, and detection of the membrane were performed using the DIG High Prime DNA Labeling and Detection kit (Roche Diagnostics, Shanghai, China) according to the manufacturer’s instructions. For the generation of RNAi strains, a 395-bp fragment from *FonPUF5* and a 377-bp fragment from *FonARP3* were amplified and cloned into the pSilent1 vector (95). The *FonPUF5*-pSilent1 and *FonARP3*-pSilent1 plasmids were introduced into fungal protoplasts and selected on PDA supplemented with Hyg. For the construction of the complementation cassette, the native promoter along with the coding region of *FonPUF1* and *FonARC18* was amplified and independently cotransformed with *Xho*I-digested pYF11-neo plasmid into the yeast strain XK1-25. The constructed vectors pYF11-*FonPUF1* and pYF11-*FonARC18* were introduced into protoplasts of respective deletion strains. Putative complemented transformants were selected on PDA supplemented with 50 μg mL^−1^ neomycin.

10.1128/mbio.00157-23.10 TABLE S3 Primers used in this study. Download Table S3, XLSX file, 0.02 MB. Copyright © 2023 Gao et al. 2023 Gao et al. https://creativecommons.org/licenses/by/4.0/ This content is distributed under the terms of the Creative Commons Attribution 4.0 International license.

### Pathogenicity tests and fungal biomass estimation assays.

Conidia were harvested from 3-day-old liquid MBL cultures and the spore concentration was adjusted to approximately 5 × 10^6^ spores mL^−1^. Two-week-old soil-grown watermelon (Citrullus lanatus L.) cv. Zaojia (a susceptible cultivar) plants were pulled out and washed gently to remove root-adhered soil particles, followed by dipping the roots in spore suspensions for 15 min. The inoculated plants were replanted in the soil and covered with plastic wrap for 3 days to maintain moisture for disease development. Disease symptoms and severity were observed and assessed during a period of 20 days postinoculation according to the following 4-scale ratings: 0 = no symptom, 1 = yellowing, 2 = wilting, and 3 = death. For *in planta* fungal growth measurement, the roots of watermelon plants were dipped in spore suspensions and grown hydroponically in a rotary shaker at 26°C and 85 rpm. The infected roots and stems were sampled at 3, 6, and 9 days postinoculation. Fungal biomass in roots or stems was measured by qPCR via analyzing the transcript levels of *FonOpm12* and watermelon *ClRps10* genes, and the relative fungal growth was calculated by normalizing fungal *FonOpm12* to watermelon *ClRps10* (51). For *Fon* growth recovery assays, the root and stem samples from inoculated plants were collected at 15 days postinoculation, sectioned into 1-cm fragments, and surface sterilized in 70% ethanol solution for 30 seconds. The root or stem fragments were placed on PDA supplemented with kanamycin or carbenicillin and incubated at 26°C for 3 days.

### Y2H assay.

Y2H assays were performed using the Matchmaker Gold Y2H System following the manufacturer’s protocol (Clontech, Mountain View, CA, USA). Briefly, coding sequences of the target genes were amplified with gene-specific primers (see Table S3) and cloned into pGBKT7 or pGADT7 vectors. The constructed vectors were cotransformed into yeast strain Y2H Gold using the lithium acetate/single-stranded DNA/PEG method (96). The pair of pGBKT7-53 and pGADT7-T was used as a positive control. Cotransformed yeast cells were grown on SD/-Leu/-Trp medium (Clontech, Mountain View, CA, USA) at 30°C for 5 days. The transformants were further screened on QDO medium (Clontech, Mountain View, CA, USA) containing 40 μg mL^−1^ X-α-Gal (Clontech, Mountain View, CA, USA) or 125 ng mL^−1^ aureobasidin A (Clontech, Mountain View, CA, USA).

### Co-IP assay.

Coding sequences of the target genes were amplified using specific primers (see Table S3) and cloned into pYF11 vector with fusion of the GFP tag at C terminus or pHZ126 vector with fusion of 3×FLAG tag (97). The fusion constructs were confirmed by sequencing and desired combinations of constructs were introduced into WT. Transformants were selected on PDA supplemented with Hyg (Roche Diagnostics, Shanghai, China) and neomycin (Sangon Biotech, Shanghai, China). Putative transformants expressing two fusion constructs were verified by PCR and Western blot analyses. Total proteins were extracted and incubated with GFP-Trap beads (ChromoTek, Planegg-Martinsried, Germany) at 4°C for 4 h. After being washed three times, proteins were eluted from the GFP-Trap beads. The eluted samples were detected by Western blotting with anti-GFP antibody (Abcam, Cambridge, MA, USA) or anti-FLAG antibody (Sigma-Aldrich, St. Louis, MO, USA).

### RNA extraction and RT-qPCR analysis.

Mycelial mass was pulverized in liquid nitrogen and total RNA was extracted using RNA isolator reagent (Vazyme Biotech, Nanjing, China) and reversely transcribed with HiScript II QRT SuperMix for qPCR (+gDNA wiper) (Vazyme Biotech, Nanjing, China). For qPCR, AceQ qPCR SYBR green Master Mix (Vazyme Biotech, Nanjing, China) was used to prepare a reaction mixture and run on a LightCycler 96 (Roche, Shanghai, China) with three technical repeats. *FonActin* was used as an internal control and relative expression of each target gene was calculated using the 2^−ΔΔCT^ method. Primers used for RT-qPCR are listed in Table S3.

### Western blotting.

Total proteins were extracted using a lysis buffer (1 M Tris-HCl, pH 7.4, 0.5 M EDTA, 1 M NaCl, 0.1% Triton X-100, 1 mM DTT, and 1× protease inhibitor cocktail; Sigma-Aldrich, St. Louis, MO, USA) and denatured with SDS loading buffer (TaKaRa, Dalian, China). The denatured protein samples were separated on 12% SDS-polyacrylamide gels (PAGE) and transferred to 0.22-μm PVDF membranes (Millipore, Billerica, MA, USA), flowed by incubation with the corresponding antibody for immunoblotting. The membrane was exposed to ECL chromogenic reagent (Thermo Fisher Scientific, Rockford, IL, USA). The Tanon automatic gel imaging system (Tianneng Corporation, Shanghai, China) was used for detecting bands and image acquisition.

### Microscopic examinations.

To detect GFP and Mito-HcRed (Beyotime Biotechnology, Nantong, China) signals, fresh mycelia were examined with a Zeiss LSM780 confocal microscope (Carl Zeiss AG, Oberkochen, Germany). To observe macroconidial septa, fresh conidia were stained with CFW and examined with the Zeiss LSM780 confocal microscope. For TEM observations, fresh mycelia were treated as described previously (82) and observed under an H-7650 TEM (Hitachi, Tokyo, Japan).

### Biochemical measurements.

ATP quantification was performed using the ATP assay kit (Beyotime Institute of Biotechnology, Shanghai, China) according to the instruction manual. Briefly, mycelia were added to a lysis buffer in the ATP detection kit. After centrifugation at 12,000 × *g* for 5 min at 4°C, the supernatant was collected for subsequent biochemical measurements. The samples were mixed with prepared ATP detection working solution and measured with the Biotek Cytation instrument (BioTek, Winooski, VT, USA). For detection of ROS in vegetative mycelia, hyphae were collected and stained with 1.5 mM DCFH-DA (Beyotime Biotechnology, Nantong, China) in 50 mM phosphate-buffered saline (PBS). After being washed twice with PBS buffer, the fluorescent signals were observed using Zeiss LSM780 confocal microscope (Carl Zeiss AG, Oberkochen, Germany). Surface plots were created using the ImageJ Software.

### EMSAs.

Prokaryotic expression and purification of recombinant GST-FonPUF1 and GST-FonPUF1-RP were performed as described previously (98). Biotin-labeled and unlabeled RNA probes were synthesized by Shanghai Jierui Biotechnology (Shanghai, China). EMSAs were performed using the LightShift Chemiluminescent RNA EMSA kit (Thermo Fisher Scientific, Rockford, IL, USA). For the binding reaction, the RNA probes were incubated with recombinant proteins in 20 μL of 1× buffer, containing 10 mM Tris-HCl (pH 7.5), 20 mM KCl, 1 mM MgCl_2_, and 1 mM DTT, at 28°C for 5 min. After incubation, the samples were separated on 4–6% PAGE gels for 1 h at 100 V using 0.5× Tris-borate-EDTA. After electrophoresis, gels were transferred to the nylon membranes (Millipore, Billerica, MA, USA) using a semidry transfer apparatus (Bio-Rad, Hercules, CA, USA). The membranes were cross-linked using the UV auto-cross-link program (UVP, Cambridge, UK), and the biotin-labeled RNA was detected according to the manufacturer’s recommendations.

### RNA-seq analysis.

WT and *ΔFonPUF1* mycelia cultured in YEPD liquid medium for 48 h were harvested for RNA-seq. Total RNA isolation and library preparation for transcriptome sequencing were performed by Novogene (Novogene, Beijing, China). Sequencing libraries were generated using NEBNext UltraTM RNA Library Prep kit for Illumina (NEB, Ipswich, MA, USA) following the manufacturer’s recommendations. Clustering of the index-coded samples was performed on a cBot Cluster Generation System using TruSeq PE Cluster kit v3-cBot-Illumina High-Seq according to the manufacturer’s instructions. After raw data quality control, reads were aligned to the reference F. oxysporum f. sp. *lycopersici* genome using HiSAT2 v2.0.5. For quantification of gene expression level, the fragments per kilobase of transcript sequence per millions base pairs (FPKM) of each gene were calculated based on the length of the gene and reads count mapped to this gene. Differential expression analysis of two groups (three biological replicates per group) was performed using the DESeq2 R package (1.16.1). GO enrichment analysis of DEGs was conducted by the clusterProfiler R package.

### Experimental design and statistical analysis.

All experiments were performed in triplicate and data are shown as mean ± standard deviation (SD) from three independent experiments. Data were subjected to statistical analysis according to the Student's *t* test or Fisher’s least significant difference (LSD) test, and the significant difference among compared data sets was calculated at a probability value of *, *P < *0.05.

### Data availability.

The transcriptome data of *Fon* WT and *ΔFonPUF1* strains were deposited in the SRA database in NCBI under accession numbers SAMN22870552, SAMN22870553, SAMN22870554, SAMN22870555, SAMN22870556, and SAMN22870557, respectively.
